# HIV-1 proteins dysregulate motivational processes and dopamine circuitry

**DOI:** 10.1038/s41598-018-25109-0

**Published:** 2018-05-18

**Authors:** Sarah J. Bertrand, Charles F. Mactutus, Steven B. Harrod, Landhing M. Moran, Rosemarie M. Booze

**Affiliations:** Behavioral Neuroscience Laboratory, Department of Psychology, University of South Carolina, Columbia, SC 29208 USA

## Abstract

Motivational alterations, such as apathy, in HIV-1+ individuals are associated with decreased performance on tasks involving frontal-subcortical circuitry. We used the HIV-1 transgenic (Tg) rat to assess effect of long-term HIV-1 protein exposure on motivated behavior using sucrose (1–30%, w/v) and cocaine (0.01–1.0 mg/kg/infusion) maintained responding with fixed-ratio (FR) and progressive-ratio (PR) schedules of reinforcement. For sucrose-reinforced responding, HIV-1 Tg rats displayed no change in EC_50_ relative to controls, suggesting no change in sucrose reinforcement but had a downward shifted concentration-response curves, suggesting a decrease in response vigor. Cocaine-maintained responding was attenuated in HIV-1 Tg rats (FR1 0.33 mg/kg/infusion and PR 1.0 mg/kg/infusion). Dose-response tests (PR) revealed that HIV-1 Tg animals responded significantly less than F344 control rats and failed to earn significantly more infusions of cocaine as the unit dose increased. When choosing between cocaine and sucrose, control rats initially chose sucrose but with time shifted to a cocaine preference. In contrast, HIV-1 disrupted choice behaviors. DAT function was altered in the striatum of HIV-1 Tg rats; however, prior cocaine self-administration produced a unique effect on dopamine homeostasis in the HIV-1 Tg striatum. These findings of altered goal directed behaviors may determine neurobiological mechanisms of apathy in HIV-1+ patients.

## Introduction

Apathy is a common motivational alteration in HIV-1+ individuals, affecting between 30–60% of the population, despite antiretroviral therapy^1,2^. Although the construct of apathy is widely used in neuropsychiatric descriptions of HIV-1^3^, apathy has received little attention in HIV-1 pre-clinical studies.

HIV-1 exerts effects on reward processes and motivation^4,5^. Interestingly, neural systems that mediate the motivational features of goal-directed behavior are damaged by HIV-1^4,6,7^. Post-mortem HIV-1+ brain tissue has high HIV-1 viral titers in the caudate^8^ and atrophy in the caudate-putamen and nucleus accumbens (NAc) are reported in HIV-1 infected patients exhibiting cognitive impairment^9^. HIV-1+ brains show alterations in dopaminergic cell bodies^10^ and loss of synaptic connectivity in the dopaminergic projection pathways^11^. Thus, the DA system in the human brain appears particularly vulnerable to HIV-1.

Motivation is a fundamental state that allows regulation of internal and external environments through the organization of behavior [e.g.^12^]. Goal-directed behavior, which encompasses Pavlovian and operant conditioning processes, is one example^13^. Once learned, goal-directed behavior is maintained by motivational and cognitive processes, such as the animals’ homeostatic state, an expectation about the reinforcer, the current value of the reinforcer, environmental cues, and other learning phenomena, such as generalization and discrimination^14,15^. However, motivational processes have received little attention in experimental models of HIV-1, despite apathy/motivational disturbances being common in the HIV-1 population^3^.

Brains from HIV-1+ patients without HIV-1 viral RNA/p24 (without active viremia, i.e., latent infections) show a reduction of synaptodendritic markers^16^ and the presence of HIV-1 Tat protein in the cerebral spinal fluid^17^. The HIV-1 transgenic (Tg) rat incorporates HIV-1 proviral DNA into its genome, without active viremia^18^. In the absence of active infection or viral replication in the HIV-1 rat, the HIV-1 proviral DNA may induce production of HIV-1 viral proteins. The HIV-1 Tat protein directly inhibits DAT via specific Tat protein:DAT protein interactions^19^. Although dopamine mediates many motivational/reward processes, and HIV-1 viral proteins interact with DAT^19,20^, it is unknown how chronic, low-levels of HIV-1 proteins (e.g., Tat, gp120) alter motivational processes and underlying neurochemical and structural responses.

Motivational alterations in HIV-1+ individuals are associated with decreased performance on tasks known to involve frontal-subcortical circuitry^21^, and are associated with decreased volume of the NAc^22^. Apathy is not generally addressed in animal models; however, the concept of motivation, or how willing an animal is to “work” for reinforcement, is frequently tested in animals using operant conditioning. In the current experiments, operant schedules of reinforcement were used to assess potential differences in sensitivity to the reinforcer^23^ and changes in reinforcement efficacy^24^ to assess motivational alterations in transgenic rats that constitutively express multiple proteins associated with the HIV-1 virus.

We used the HIV-1 Tg rat to examine alterations in motivational processes and dopaminergic neurochemical correlates in the striatum and prefrontal cortex. Specifically, we investigated goal-directed behavior, using self-administration of sucrose and/or IV cocaine as reinforcement, and neurochemical alterations in the dopaminergic system. Regardless of whether sucrose and/or IV cocaine self-administration is specifically affected by HIV-1 proteins, these findings would help elucidate mechanisms of motivational alterations, such as apathy, in HIV-1+ patients.

## Materials and Methods

### Animals

Ovariectomized female HIV-1 Transgenic F344/NHsd rats (*n* = 14) and female F344/NHsd control rats (*n* = 15) were procured at approximately 60 days of age from Harlan Laboratories, Inc. (Indianapolis, IN, USA). The generation of the HIV-1 Tg rat has been previously described^18^. In brief, the hemizygous HIV-1 Tg animals were produced using a construct derived from an infectious provirus (pNL4–3) after deletion of a 3.1 kb *Sph1-Bal1* fragment that overlaps the *gag* and *pol* genes (pNL4-3d1443). Thus, the HIV-1 Tg rats produce Tat and gp120 proteins^18^, but not Gag or Pol proteins, and therefore, these animals are non-infectious. Constitutive HIV-1 protein expression in the HIV-1 Tg rat is under the control of the natural HIV-1 promoter, LTR, with HIV-1 protein expression in mononuclear phagocytes^25^ astrocytes and possibly other cells^26^. Due to the potential for sporadic transgene insertion elsewhere in the rat genome, which might have untoward effects, normal F344/NHsd controls are used (rather than littermates) to assure the most developmentally appropriate and genetically stable baseline. Age-matched F344/NHsd controls were contemporaneously purchased from the same supplier; the HIV-1 Tg rat is on the same F344/NHsd background.

All rats were ovariectomized (OVX) at Harlan Laboratories prior to arrival at the University of South Carolina. Ovariectomy was performed as estradiol protects against HIV-1 mediated neuronal damage *in vitro*^27,28^. In addition, rats were fed a minimal phytoestrogen diet (≤20 ppm of phytoestrogen; Teklad 2020X Global Rodent Diet; Harlan Laboratories, Inc., Indianapolis, IN). Phytoestrogens are plant-derived compounds that are structurally similar to estrogen, have estrogenic effects, and are found in soybean and alfalfa meal^29^. Soy and alfalfa meal are typical components of rodent chow; standard rodent chow typically contains 200–500 ppm (Harlan Laboratories, Inc., USA). Food and water were provided *ad libitum* throughout the experiment, unless otherwise specified. The targeted conditions for maintenance of the animal vivarium were a temperature of 20 ± 2 °C, 50 ± 10% relative humidity and a 12 L:12D cycle with lights on at 0700 h EST. The protocol for this research methodology was approved by the Institutional Animal Care and Use Committee (IACUC) at the University of South Carolina (animal assurance number: A3049-01). All animal procedures were performed in accordance with this federal assurance.

### Apparatus

#### Sucrose preference experiment

Animals received access to water or sucrose solutions in 100 ml graduated cylinders equipped with a # 6.5 rubber stopper and 2.5” straight drinking tube (OT-100; Ancare, Bellmore, NY, USA) that was placed on the testing cage. Animals were tested in clear polycarbonate cages containing standard rodent bedding. Five 100 ml graduated cylinders, containing four sucrose solutions or water, were fitted to the cage-top of the rats’ testing cage. The testing cages were located on a single cage rack and the experiment was conducted in the colony room.

#### Operant conditioning experiments

The operant chambers (ENV-008; Med-Associates, St. Albans, VT, USA), were housed within sound-attenuating enclosures and controlled by Med-PC computer interface software. The front and back panels of the chamber were stainless steel panels, the sides and top of the chamber were constructed of polycarbonate. The front panel of the chamber housed a panel containing a receptacle that allows a recessed 0.01 cc dipper cup (ENV-202C) to deliver a solution through a 5 cm × 5 cm opening following completion of a response requirement (ENV 202M-S). Two retractable metal levers (ENV-112BM) on either side of the receptacle were located 7.3 cm above a metal grid floor. These were “active” levers (responding on them could result in reinforcement). A 28-V white cue light, 3 cm in diameter, was located above each active response lever but never illuminated. An infrared sensor (ENV-254-CB) was used to detect head entries into the receptacle. An additional non-retractable lever was positioned on the back wall of the chamber and a 28-V house light was located above the lever. This was the “inactive” lever and there were no programmed response-reinforcer contingencies with the lever. During cocaine self-administration tests a syringe pump (PHM-100) was used to deliver intravenous drug infusions through a water-tight swivel (Instech 375/22 ps 22 GA; Instech Laboratories, Inc., Plymouth Meeting, PA), which was connected to the back mount of the animal using Tygon tubing (ID, 0.020 IN; OD, 0.060 IN) enclosed by a stainless steel tether (Camcaths, Cambridgeshire, GB). The pump infusion times were calculated by a Med-PC computer program according to the animal’s daily bodyweight.

### Drugs

Cocaine hydrochloride (Sigma-Aldrich Pharmaceuticals, St. Louis, MO) was weighed as the salt and was dissolved in physiological saline (0.9%; Hospira, Inc. Lake Forest, IL). Cocaine and sucrose solutions were prepared fresh prior to the start of each session to prevent any significant hydrolysis of cocaine and to ensure that each group received similar exposure to either solution prior to each operant test. Heparin was purchased from APP Pharmaceuticals (Schaumburg, IL.), butorphenol (Dolorex) from Merck Animal Health (Millsboro, DE), sevofluorane, USP from Baxter (Deerfield, IL), and gentamicin sulfate from VEDCO (Saint Joseph, MO).

### Synaptosomal [^3^H]DA Uptake Assay and [^3^H]WIN35,428 Binding

D-Glucose was purchased from Aldrich Chemical Co, Inc. (Milwaukee, WI). [^3^H]DA (3,4-ethyl-2[*N*-^3^H] dihydroxyphenylethylamine; specific activity, 31 Ci/mmol), and [^3^H]WIN 35,428 (−)-3β-(4-flurophentl)-tropan-2β-carboxylic acid methyl ester tartrate; specific activity, 85 Ci/mmol) were purchased from PerkinElmer Life Sciences (Boston, MA). L-Ascorbic acid, bovine serum albumin, pyrocatechol, α-D-glucose, HEPES, nomifensine maleate, desipramine hydrochloride, paroxetine hydrochlorine, pargyline hydrochloride, and sucrose were purchased from Sigma Aldrich (St. Louis, MO). All other chemicals were purchased from Fisher Scientific (Waltham, MA).

### Timeline and Overall Experimental Design

A two-group between-subjects design was employed (HIV-1 Tg vs. F344/NHsd). Animals were successively assessed, trained, and tested for approximately six months, as illustrated in the design table (Table 1). After completion of the first phase with sucrose reinforcement, four of each of the F344 rats and HIV-1 Tg rats were set aside to be used as controls for the cocaine-induced dopamine transporter studies in phase four. During phases two and three these particular control animals never received any cocaine reinforcement, but were otherwise treated similarly, i.e., they were catheterized and maintained on 5% sucrose reinforcement.

**Table 1:** Overall Experimental Design.

Phase 1: Sucrose Reinforcer				
Sucrose Preference	Self-Administration FR Acquisition: Restricted	Self-Administration FR Acquisition: Non-Restricted	Self-Administration FR-Dose Response	Self-Administration PR-Dose Response
Phase 2: Cocaine Reinforcer				
Self-Administration FR Acquisition	Self-Administration PR-Escalation			Self-Administration PR-Dose Response
Phase 3: Choice Between Reinforcers				
Self-Administration FR Choice	Self-Administration Saline Substitution			
Phase 4: Dopamine Transporter				
Synaptosomal [^3^H]DA Uptake	[^3^H]WIN35,428 Binding			

### Experiment 1: Sucrose Taste Preference

A sucrose taste preference test was conducted to assess HIV-1 Tg rats’ preference for one of five concentrations of sucrose, i.e., 0, 1, 3, 10, and 30% (w/v), relative to F344 controls. In order to habituate the rats to the novel bottles and stoppers, rats were placed in a clean testing cage with access to five graduated cylinders that contained water. The habituation procedure occurred for 30 min per day for 3 consecutive days. The sucrose preference tests were conducted for five consecutive days following habituation. During testing animals were placed in a clean testing cage and had free access to the sucrose solutions and water for 60 min. To control for a position bias, bottle order was randomized for each rat on the first day of testing and bottles were rotated on each subsequent day using a Latin square procedure so that each concentration was assessed in each location. Bottles were weighed and menisci read to the nearest ml before and after each test to determine the amount of fluid consumed from each bottle.

### Experiment 2: Sucrose-Maintained Responding

The animals from Experiment 1 were used in the subsequent experiments, and thus all animals were sucrose experienced. Dipper training and auto-shaping were conducted according to previous research^30^. All rats learned to approach the receptacle and drink from the dipper (dipper training) and subsequently respond for 5% sucrose (w/v) reinforcement (autoshaping). Rats were water restricted for 12–15 hr prior to dipper training, autoshaping, and fixed ratio (FR) training. After completion of the daily operant test, animals were given free access to water in their home cage for 9–12 hr.

Fixed-ratio (FR) schedules of reinforcement provide the reinforcer following a fixed set of responses. In the case of drug reinforcement, responding changes if the unit-dose of the self-administered drug is varied^31^. In order to assess the reinforcing efficacy of a food or drug, a progressive-ratio (PR) schedule of reinforcement is often used^32^. According to the PR schedule, the number of responses required to produce reinforcement is increased following completion of each ratio requirement. The greater the responding, the more reinforcement is provided by a particular stimulus, such as sucrose or cocaine^30,32,33^.

Two successive experiments were conducted to investigate acquisition of sucrose-maintained responding. Experiments 2A and 2B investigated sucrose-maintained responding under conditions of water restriction and non-restriction (*ad libitum* water), respectively. In both experiments, animals were required to respond for 5% sucrose according to an FR1 schedule of reinforcement. The FR1 schedule requires one response on an active lever to receive the consequent reinforcer; hence, the ratio requirement is fixed. At the beginning of the operant test the active and inactive levers were available. Rats responded for sucrose reinforcement (5%, w/v) on the two active levers during 42-min operant tests. A single response on an active lever resulted in retraction of both levers during the 4 sec of access to sucrose. In order to prevent the development of a response side bias, if an animal made five consecutive responses on either one of the active levers, that lever was programmed to retract. If a lever was retracted, the alternative lever was available for responding. Once the asymmetry in responding was balanced the previously retracted lever was once again made available. The use of two active levers was also important from the perspective of training the animals about response variability^34^, especially in light of the subsequent assessment of choice behavior (Experiment 4). Responding on the inactive lever was recorded, but no programmed consequence was presented. A house-light, located above the inactive lever, was illuminated throughout the entire experiment. No time-out periods were used in experiments 2A or 2B. Animals were required to meet a criterion of 60 or more reinforcers per test over 3 consecutive days. Once this criterion was met under conditions of water restriction (2A), the experiment was conducted again under conditions of *ad libitum* water (2B). No further water restriction was used for any subsequent experiment.

Experiment 2C determined the sucrose-maintained responding curves for the F344 or HIV-Tg rats using the FR1 schedule of reinforcement. A single response on an active lever resulted in retraction of both levers during the 4 sec of access to sucrose. Testing began after three consecutive days of 60 or more reinforcers using the training concentration of sucrose (5%; FR1). For the tests, one of five sucrose concentrations (1%, 3%, 5%, 10%, and 30%; w/v) was presented, according to a Latin-square procedure. There were six total concentration-response test days and those occurred every other day. Maintenance trials occurred on the non-test days. The training concentration of sucrose (5%) was the reinforcer on the maintenance days. The house-light was illuminated throughout the entire experiment, and no time-out periods were used in experiment 2C. Water was the reinforcer on the sixth and final test day. Water was used primarily as a control condition to assess responding in the absence of sucrose. It was presented as the sixth and last reinforcer for all rats in order to prevent potential extinction learning if water was not reinforcing to the animals (e.g., the absence of reinforcement).

Experiment 2D determined the sucrose-maintained responding curves for the F344 or HIV-Tg rats using the progressive-ratio (PR) schedule of reinforcement. Animals were experienced with FR schedules and did not require new training in order to progress to the PR phase of the experiment. After three maintenance days (5%; FR1) the schedule of reinforcement was changed to PR. During PR tests completion of the ratio requirement resulted in retraction of both levers during the 4 sec of access to sucrose. A house-light was illuminated throughout the entire experiment, and no time-out periods were used in experiment 2D. Ratios progressively increased according to an exponential function (Response ratio (rounded to nearest integer) = [5e^(injecti^°^n number × 0.2)^] − 5)^32^.

The combination of responses across the two active levers that completed the ratio requirement provided delivery of reinforcement. One of five sucrose concentrations (1%, 3%, 5%, 10%, and 30%; w/v; Latin-square procedure) was presented as the reinforcer on the concentration-response test days, which occurred every other day. Maintenance trials, during which 5% sucrose was available (FR1), occurred on the non-PR test days. Water was presented as the sixth and last reinforcer for all rats in order to prevent potential extinction learning if water was not as reinforcing to the animals. The PR tests were a maximum of 120 min in length. No water restriction was used for this experiment.

### Experiment 3: Cocaine-Maintained Responding

#### Surgery

Following completion of experiment 2D, all animals, including the neurochemical controls, were implanted with IV catheters. IV catheterization was performed according to established methods^35^. Anesthesia was induced using 5–7% inhalant sevofluorane and animals were maintained at 3–4% sevofluorane throughout the surgical procedure. Following anesthesia, a sterile IV catheter was implanted into the right jugular vein and secured with 4-0 Perma-Hand Silk sterile sutures (EthiconEndo-Surgery, Inc). The dorsal portion of the catheter was affixed to an acrylic pedestal embedded with mesh. The dorsal portion/backmount was subcutaneously implanted above the right and left scapulae, and then sterile 4-0 Monoweb sutures were used to stitch the backmount into place. All rats were administered butorphenol (1.0 mg/kg, s.c.) and gentamicin (0.2 ml 1%, i.v.) immediately following the surgery to provide post-operative analgesia and to prevent infection, respectively. Immediately after surgery, animals were monitored in a heat-regulated warm chamber and returned to the colony room following recovery from anesthesia. All animals survived surgery. The catheters were “flushed” daily with a post-flush solution containing the anti-coagulant heparin (2.5**%**) and 0.2 ml gentamicin (1%) for an additional 7 days to prevent blood clotting and infections following the surgery. Prior to testing each day, catheters were flushed with a 0.9% saline solution, and following the operant test catheters were treated with the post-flush solution. Cocaine testing began at least 7 days post-surgery for the HIV-1 Tg and F344 rats that received cocaine reinforcement in this experiment. The HIV-1 Tg and F344 neurochemical controls received sucrose (5%, w/v) reinforcement instead of cocaine, but.were connected to the back mount via the tether that delivers drug, exactly like that of the animals receiving cocaine reinforcement.

#### Cocaine-maintained responding

In brief, the cocaine self-administration procedure was a modified version as previously published^24^. In our experiments, rats responded for cocaine according to a FR1 schedule of reinforcement, and were switched to daily PR tests with a significantly higher concentration of IV cocaine. Morgan *et al*.^24^ showed that the reinforcing efficacy of cocaine increased over the 14 PR test days, as rats demonstrated a progressive increase in cocaine-maintained responding. We used this procedure to assess potential differences in cocaine-maintained responding between the HIV-Tg and F344 animals.

There were three segments to experiment 3. In phase one, HIV-Tg and F344 rats could respond for cocaine (0.33 mg/kg/infusion) according to a FR1 schedule of reinforcement. Segment one lasted for five days and each operant test was one hour in duration. In segment 2, animals could respond for cocaine (1.0 mg/kg/inf) according to a PR schedule of reinforcement (Response ratio (rounded to nearest integer) = [5e^(injection number × 0.2)^] − 5)^32^, and this segment lasted for 14 days. Daily operant tests were a maximum of 120 min. The program timed out if 60 min elapsed without the completion of the next response requirement. In the final segment, a PR cocaine dose-response experiment was conducted. The cocaine concentrations were 0.01, 0.03, 0.10, 0.33, and 1.0 mg/kg/inf and they were presented in an ascending manner. During segments 1–3, completion of the response requirement resulted in the retraction of the active levers and the extinction of the houselight for 20 sec. Responses on the inactive lever were recorded, but no programmed consequence was presented. Maintenance trials, which made the 0.33 mg/kg/inf cocaine concentration available on an FR1 schedule, occurred every other day.

### Experiment 4: Choice Behavior

Following completion of the cocaine studies, both groups of animals were subject to a choice self-administration protocol. A FR1 schedule of reinforcement was used in the choice experiments. The EC_50_ for sucrose and cocaine were used as the concentrations for choice behavior. The EC_50_ for the F344 controls was the training dose (0.33 mg/kg/infusion) and due to a lack of differential responding in the HIV-1 Tg animal the training dose was also used as the test dose. Each session began with 4 forced-choice trials (2 nondrug and 2 drug rewards) followed by the choice component. During forced-choice, only one active lever was present. The position of the sucrose paired lever (right or left) was balanced between groups. During the choice component, both levers were available simultaneously allowing the animals to freely choose between sucrose and cocaine. Following a response, the active levers retracted and the house light was turned off for 20 sec. There was an inactive lever located in the center back panel of the chamber. Responding on the inactive lever was recorded, but no programmed consequence was presented. Saline was substituted for cocaine on day 8 of the choice self-administration procedure. The HIV-1 Tg and F344 neurochemical controls received sucrose (5%, w/v) reinforcement, rather than cocaine reinforcement.

### Experiment 5: Neurochemical measures of the dopamine system

#### [^3^H] DA uptake in striatum and PFC

Animals were sacrificed 12–18 hrs following completion of the self-administration choice procedure. The kinetic parameters (*V*_max_ and *K*_m_) of synaptosomal [^3^H]DA uptake were determined using a previously described method^19^. Briefly, brain regions from each rat were homogenized following sacrifice in 20 ml of ice-cold sucrose solution (0.32 M sucrose and 5 mM sodium bicarbonate, pH 7.4) with 16 up-and-down strokes using a Teflon pestle homogenizer. The resulting crude synaptosomal preparation was centrifuged at 2,000 *g* for 10 min at 4 °C, and the subsequent supernatants were centrifuged at 20,000 *g* for 15 min at 4 °C. These pellets were then resuspended (4.0 ml for PFC pellets and 3.0 ml for striatal pellets) in ice-cold Krebs-Ringer-HEPES assay buffer (125 mM NaCl, 5 mM KCl, 1.5 mM MgSO_4_, 1.25 mM CaCl_2_, 1.5 mM KH_2_PO_4_, 10 mM D-glucose, 25 mM HEPES, 0.1 mM EDTA, 0.1 mM pargyline, and 0.1 mM L-ascorbic acid, saturated with 95%O_2_/5%CO_2_, pH 7.4). Half of the striatal synaptosomes from individual rats were used in the [^3^H]DA uptake assay, and the other half were used for the [^3^H]WIN 35,428 binding assays. In order to isolate the uptake of DA by DAT in the PFC, kinetic analysis of [^3^H]DA uptake by the DAT in the PFC was assessed in the presence of desipramine (1 µM) and paroxetine (5 nM) to prevent [^3^H]DA uptake into norepinephrine and serotonin containing nerve terminals. The Bradford protein assay, using bovine serum albumin as the protein standard, was used to determine protein concentrations (BioRad, USA).

Synaptosomes containing ~100 µg protein/100 µl (PFC) and ~50 µg protein/30 µl (striatum) were incubated in a metabolic shaker for 10 min at 34 °C and then incubated for 8 min at 34 °C after adding one of 8 [^3^H]DA concentrations (1 nM - 1 µM, final concentration). Assays were performed in duplicate with a total volume of 500 µl. Incubation was terminated by the addition of 3 ml ice-cold assay buffer. Samples were immediately filtered through Whatman GF/B glass fiber filters using a Brandel cell harvester (Model M-48 Biochemical Research and Development Laboratories Inc., Gaithersburg, MD). Filters were presoaked with 1 mM pyrocatechol in assay buffer and washed 3 times with 3 ml ice-cold assay buffer containing pyrocatechol. Pyrocatechol, a COMT inhibitor, was included in the DA uptake assay buffer to prevent the degradation of [^3^H]DA during the processes of washing and harvesting. Non-specific uptake was determined, in duplicate, at each [^3^H]DA concentration by including 10 µM nomifensine in the assay buffer. Radioactivity was determined by liquid scintillation spectrometry (Model TRI-CARB 2900TR, Perkin Elmer Instruments, Shelton, CT, USA). GraphPad Prism 5.0 (GraphPad Software, Inc., San Diego, CA, USA) was used to determine kinetic parameters (*V*_max_ and *K*_m_).

#### Striatal [^3^H]WIN 35,428 Binding Assay

In order to determine whether [^3^H]DA uptake into striatal synaptosomes was the result of a direct interaction with the DAT protein, the maximal number of binding sites (*B*_max_) and affinity (*K*_d_) for [^3^H]WIN 35,428 binding in striatal synaptosomes were examined using a previously described method^19^. Therefore, half of the striatal synaptosomes from each rat were centrifuged at 20,000 *g* for 15 min at 4 °C and resuspended in 3.0 ml ice-cold sodium-phosphate buffer (2.1 mM NaH_2_PO_4_, 7.3 mM Na_2_HPO_4_7H_2_O, pH 7.4).

To generate saturation isotherms, striatal synaptosomes containing ~50 µg/30 µl were added to the assay tubes containing one of the eight concentrations of [^3^H]WIN 35,428 (0.5 to 30 nM, final concentration). Assay tubes were incubated for 2 hr on ice. Nonspecific binding at each concentration of [^3^H]WIN 35,428 in the presence of cocaine (30 µM, final concentration) was subtracted from the total binding to calculate the specific binding. Rapid filtration (Brandel cell harvester) onto Whatman GF/B glass fiber filters, which were presoaked for 2 hr with assay buffer containing 0.5% polyethylenimine, was used to terminate the assay. Filters were rinsed 3 times with 3 ml ice-cold assay buffer. Radioactivity on the filters was determined using liquid-scintillation spectrometry (Model TRI-CARB 2900TR, PerkinElmer Instruments, Shelton, CT, USA).

### Data analysis

Data analyses were performed using SPSS version 21 (IBM Corp., Somers, NY), BMDP version 2009 (Statistical Solutions, Saugus, MA), and GraphPad Prism version 5.02 (GraphPad Software, Inc. La Jolla, CA). For experiment 1, a mixed-design analysis of variance (ANOVA) was used to analyze sucrose consumption, with genotype (HIV-1 Tg vs. F344 control) as the between-subjects factor, and sucrose concentration (0–30%, w/v, or day 1–5) as the within-subjects factor. Experiments 2A and 2B were analyzed using an independent samples t-test, using time to meet criterion (60+ reinforcers over 3 days) as the measure of performance. Experiments 2C and 2D were analyzed using a mixed-design ANOVA with genotype (HIV-1 Tg vs. F344 control) as the between-subjects factor and sucrose concentration as the within-subjects factor. Similarly, for experiments 3A-C, a mixed-design ANOVA was used and genotype was the between-subjects factor and day or concentration was the within-subjects factor. For experiment 4, a 2 (genotype) × 2 (reinforcer type) mixed-design ANOVA was used to analyze the slope of the linear regression lines representing the change in sucrose and cocaine preference over the first 5 days of the choice period (slope values were obtained for each animal for each reinforcer from linear regression analyses on the number of reinforcers obtained over the first 5 days of the choice period). A 2 (genotype) × 2 (reinforcer type) × 5 (day) mixed-design ANOVA was used to analyze number of reinforcers earned and number of active lever press responses. Greenhouse-Geisser corrections were used in cases where Maulchey’s tests detected a violation of sphericity.

For experiment 5, which assess DA uptake and binding, the data are presented as mean values ±S.E.M.; *n* represents the number of independent experiments for each treatment group. Kinetic parameters of [^3^H]DA uptake (*V*_max_ and *K*_m_) and [^3^H]WIN 35,428 binding (*B*_max_ and *K*_d_) were determined from saturation curves by nonlinear regression analysis using a one-site model with variable slope. To determine the differences in the kinetic parameters, separate two-way ANOVAs were used to evaluate any differences between genotype (HIV-1 Tg vs. F344 control) and treatment (cocaine or sucrose) between-subject factors. Simple pairwise comparisons were used to evaluate planned-comparisons. Log transformed values of *K*_m_ or *K*_d_ were utilized for statistical comparisons. Differences were considered significant at *p* ≤ 0.05.

Stepwise regression was used to detect any significant behavioral predictors of neurochemical changes observed in the DA uptake and binding experiments. Significant models were further explored using multiple linear regression analyses. Models were considered significant if *p* ≤ 0.05.

## Results

### The HIV-1 Tg rat does not exhibit altered sucrose taste preference

**Figure 1:**
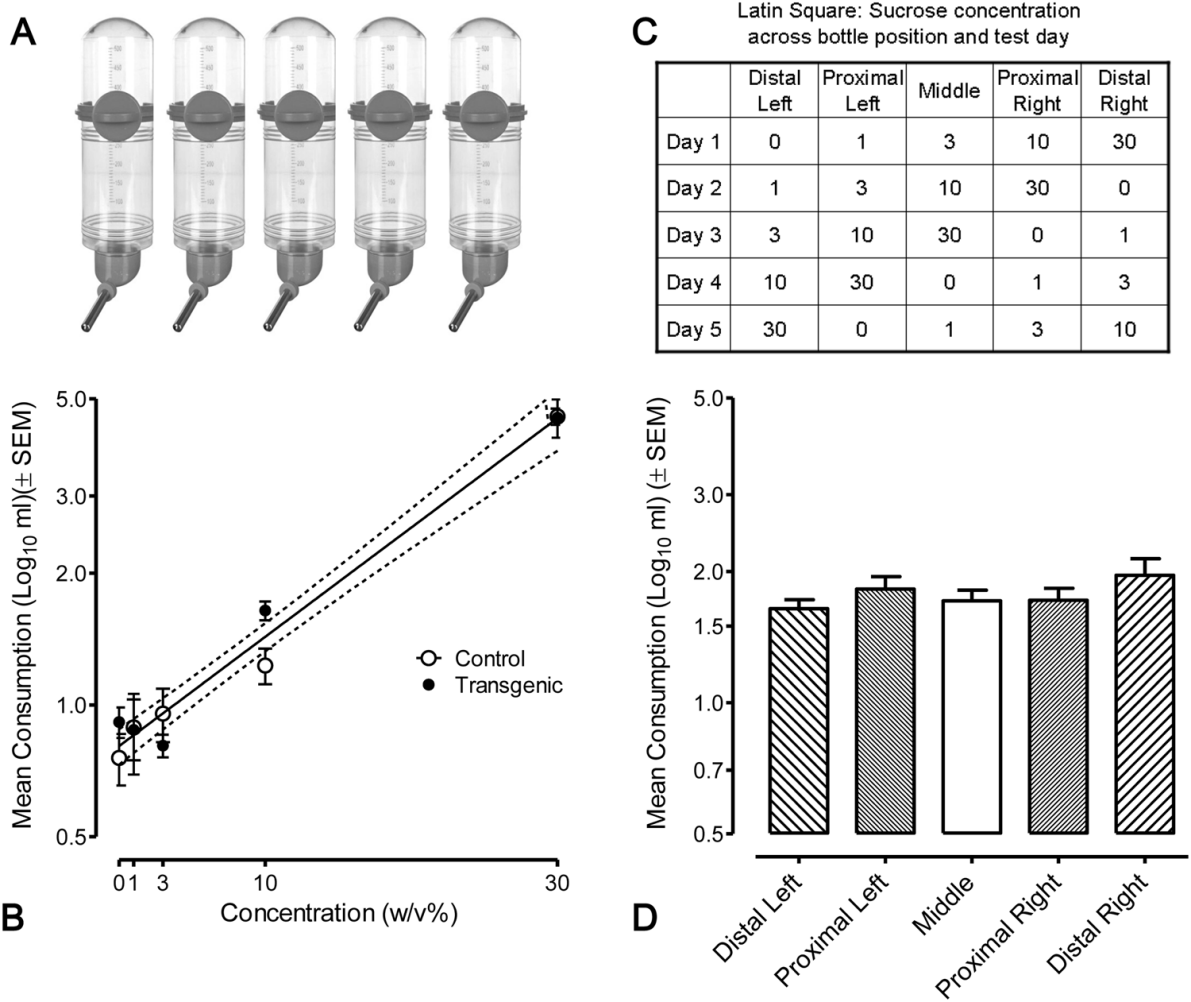
The HIV-1 Tg rat does not exhibit altered sucrose preference. Prior to evaluation of the effects of long-term HIV-1 protein exposure on motivated behavior using sucrose, an assessment of sucrose preference was conducted. (**A**) Animals had free access for 1 hour to five bottles filled with 0, 1, 3, 10, or 30% (w/v) sucrose solutions; bottles were weighed and menisci read to the nearest ml before and after each test. (**B**) Mean (±95% CI) consumption (ml) of each solution over the 5 testing days illustrated that regardless of genotype, fluid consumption was a robust linear function of sucrose concentration [*F*(1,27) = 81.8, *p* ≤ 0.001]. (**C**) To control for a position bias, bottle order was randomized for each rat on the first day of testing and bottles were rotated on each subsequent day using a Latin square procedure so that each concentration was in each location once. (**D**) Mean (±SEM) fluid consumption from each bottle position across days revealed there was no confound with any positional or side bias of the animals, i.e., there was no significant effect of bottle position, day, or a day x position interaction. (n = 14, HIV-1 transgenic F344/NHsd and *n* = 15 F344/NHsd controls).

We first examined the ability of HIV-1 Tg rats to detect sucrose and whether any taste preference might bias subsequent testing of the HIV-1 Tg rats using sucrose as a reinforcer (Fig. 1A). A 2 × 5 × 5 mixed-design ANOVA revealed a significant main effect of concentration [*F*(4, 108) = 55.0, *p*_GG_ ≤ 0.001], and a significant day × concentration interaction [*F*(16, 432) = 3.1, *p*_GG_ ≤ 0.05]. There was no significant main effect of genotype [*F*(1,27) < 1.0], nor an interaction of genotype x concentration [*F*(4, 108) = 1.3, *p* > 0.25]. As illustrated (Fig. 1B), fluid consumption was a robust linear function of sucrose concentration from 0–30% w/v [linear component, F(1,27) = 81.8, *p* ≤ 0.001]. Sucrose preference was not altered in the HIV-1 Tg rats, relative to F344 controls.

Critically, bottle position was also evaluated in order to assess any potential confound with animal side bias or preference, as illustrated in the Latin-Square design (Fig. 1C). A 2 × 5 × 5 mixed-design ANOVA of bottle position revealed no significant main effect of bottle position, day, or day × position interaction (Fig. 1D) indicating that the sucrose taste preference results were not altered by any positional or side bias of the animals.

### HIV-1 Tg rats successfully acquire sucrose-maintained responding under either restricted or non-restricted conditions

**Figure 2:**
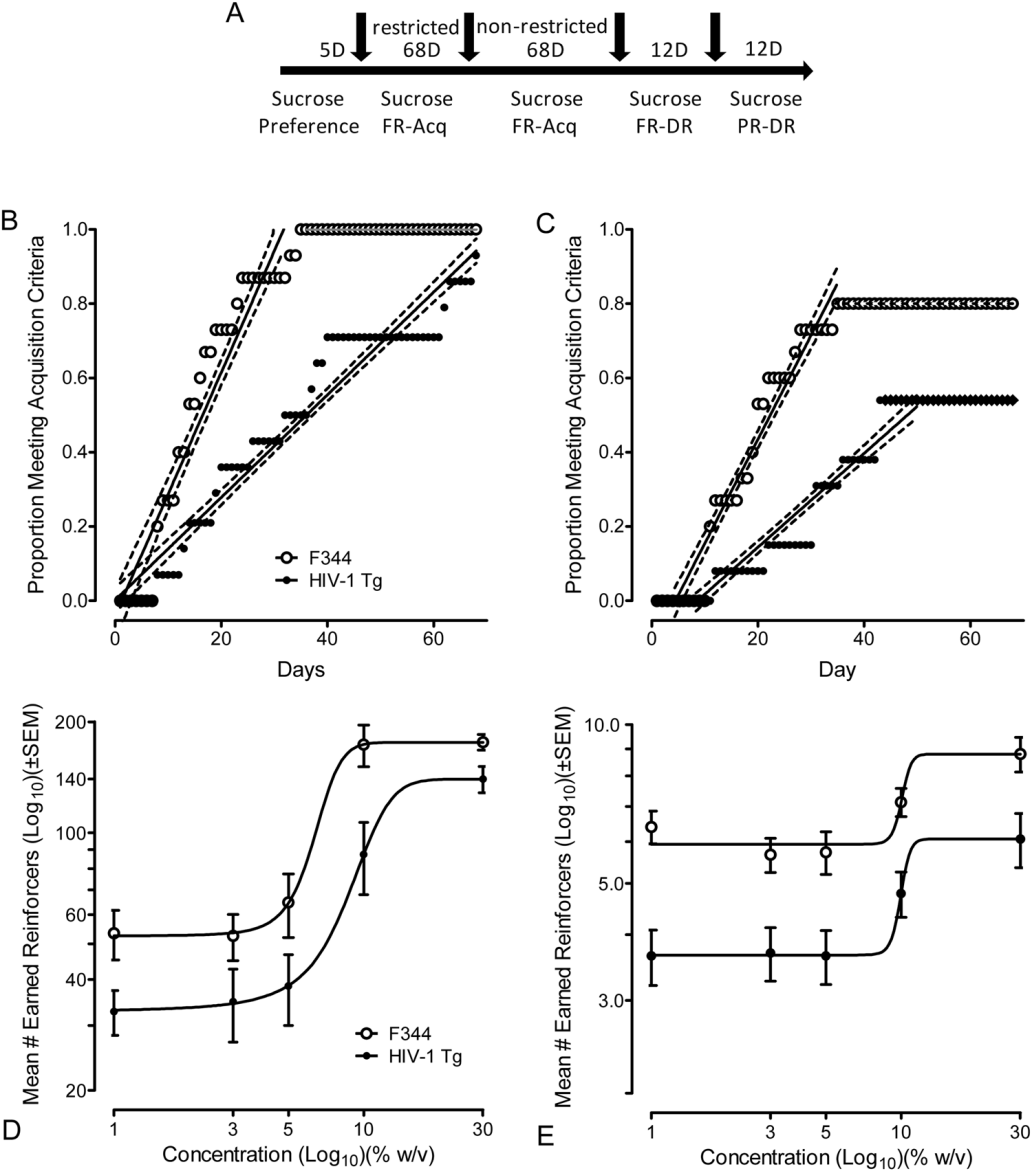
Response vigor for, but not sensitivity to, or reinforcing efficacy of, sucrose reinforcement was diminished in the HIV-1 Tg rat across multiple sucrose concentrations. (**A**) Given that there were no alterations in sucrose preference, operant procedures were used to determine sensitivity to sucrose reinforcement. (**B**) HIV-1 Tg rats successfully acquired (criterion of 60+ reinforcers for 3 consecutive days) sucrose-maintained responding under conditions of 12–15 hr of water restriction. No overall effect of the HIV-1 transgene on the number of animals that were able to acquire the task was detected after 68 days (15/15 vs. 13/14, F344 controls and the HIV-1 Tg rats, respectively), however, the HIV-1 Tg animals took longer to reach criterion relative to F344 controls (slopes of the lines were significantly different from one another [*F*(1,99) = 177.1 *p* ≤ 0.001]). (**C**) HIV-1 Tg rats successfully acquired (criterion as above) sucrose-maintained responding without extrinsic motivation (no water restriction). No overall effect of the HIV-1 transgene on the number of animals that were able to acquire the task was found at the end of 68 days (12/15 vs. 7/13, F344 controls and the HIV-1 Tg rats, respectively]. Again, however, the HIV-1 Tg animals took longer to reach criterion, relative to F344 controls (slopes of the lines were significantly different) [*F*(1,74) = 193.3, *p* ≤ 0.001]). (**D**) A robust characterization of the dose-response function (1–30% w/v sucrose) under the FR schedule of reinforcement was provided for both HIV-1 Tg and control animals by a sigmoidal curve fit (*r*^2^ = 0.99 for both the HIV-1 Tg and F344 control groups). The curve was shifted significantly downward [*F*(4,2) = 1194.0, *p* ≤ 0.001], however, there was no significant shift in the EC_50_, providing no compelling evidence for any change in sensitivity to sucrose as a reinforcer in the HIV-1 Tg animals. (**E**) Similarly, a robust characterization of the dose-response function (1–30% w/v sucrose) under the PR schedule of reinforcement was provided for both HIV-1 Tg and control animals by a sigmoidal curve fit (*r*^2^ = 0.99 and *r*^2^ = 0.95 for HIV-1 Tg and F344 controls, respectively). The curve was significantly shifted downward [*F*(3,4) = 57.4, *p* ≤ 0.001]; however, again, there was no significant shift in the EC_50_, providing no compelling evidence for any change in efficacy of sucrose as a reinforcer in the HIV-1 Tg animals. (n = 14, HIV-1 transgenic F344/NHsd and *n* = 15 F344/NHsd controls).

Given that there were no alterations in sucrose preference, operant procedures were used to determine sensitivity to sucrose reinforcement (Fig. 2A). The proportion of animals (HIV-1 Tg or F344 controls) meeting criterion across days under conditions of water restriction, as illustrated (Fig. 2B) showed there was no overall effect of the HIV-1 transgene on the number of animals that were able to acquire the task; by the end of 68 days, 100% of the F344 controls and 93% of the HIV-1 Tg rats met criterion (15/15 vs. 13/14, z = 1.1, *p* ≤ 0.29). However, also illustrated is the observation that the HIV-1 Tg animals took longer (a mean of 20 additional days) to reach criterion (60+ reinforcers for 3 consecutive days) relative to F344 controls. A linear regression on the proportion data revealed that the results for both genotypes were a significantly linear fit (*r*^2^ = 0.92 and *r*^2^ = 0.94 for F344 and HIV-1 Tg animals, respectively) and further confirmed that the slopes of the lines were significantly different from one another [*F*(1,99) = 177.1 *p* ≤ 0.001].

Once animals met criterion for 3 consecutive days under water restriction, they were allowed *ad libitum* access to water in their home cages and were tested on an FR1 schedule of reinforcement. The proportion of animals (HIV-1 Tg or F344 controls) meeting criterion across days without extrinsic motivation (no water restriction), as illustrated (Fig. 2C), showed there was no overall effect of the HIV-1 transgene on the number of animals that were able to acquire the task; by the end of 68 days, 80% of the F344 controls and 54% of the HIV-1 Tg rats met criterion [12/15 vs. 7/13, z = 1.5, *p* ≤ 0.14]. Again, also illustrated is the observation that the HIV-1 Tg animals took longer (a mean of 20 additional days) to reach criterion (60 + reinforcers for 3 consecutive days), relative to F344 controls. Specifically, regression on the proportion of animals meeting criterion across days revealed robust linear functions (*r*^2^ = 0.95 and *r*^2^ = 0.89 for F344 and HIV-1 Tg animals, respectively) and confirmed that the slopes of the lines were significantly different from one another [*F*(1,74) = 193.3, *p* ≤ 0.001].

Thus, HIV-1 Tg rats successfully acquired the self-administration task, under either restricted or even non-restricted conditions, albeit the rate at which the animals acquired the task was significantly slower as a function of expression of the HIV-1 transgene.

### Response vigor for, but not sensitivity to, or reinforcing efficacy of, sucrose reinforcement was diminished in the HIV-1 Tg rat across multiple sucrose concentrations

Next, a FR schedule of reinforcement was used to determine if there were any detectable differences in sensitivity to sucrose as a reinforcer across multiple concentrations of sucrose. A 2 × 6 repeated-measures ANOVA on total earned reinforcers revealed a main effect of genotype [*F*(1,27) = 10.7 (*p* ≤ 0.003)], a main effect of concentration [*F*(5, 135) = 65.9, *p*_GG_ ≤ 0.001], and more importantly, a genotype x concentration interaction [*F*(5,135) = 5.2, *p*_GG_ ≤ 0.006]. More specifically, as illustrated (Fig. 2D), a sigmoidal curve fit provided a robust characterization of the dose-response function for both HIV-1 Tg and control animals (*r*^2^ = 0.99 for both the HIV-1 Tg and F344 control groups). The curve was shifted significantly downward [*F*(4,2) = 1194.0, *p* ≤ 0.001], however, there was no significant shift in the EC_50_; i.e., although there was clear evidence of a decrease in overall response vigor as suggested by the overall decrease in responding, there was no compelling evidence for any change in sensitivity to sucrose as a reinforcer in the HIV-1 Tg animals.

A PR schedule of reinforcement was then used to assess potential differences in reinforcement efficacy of sucrose across a range of concentrations [i.e., PR;^24^]. A 2 × 6 repeated-measures ANOVA on total earned reinforcers revealed a main effect of genotype [*F*(1,27) = 18.5, *p* ≤ 0.001], and a main effect of concentration [*F*(5,135) = 9.1, *p*_GG_ ≤ 0.001] but no significant concentration x genotype interaction. A sigmoidal dose-response analysis, as illustrated (Fig. 2E), again confirmed a robust characterization of the dose-response function for both HIV-1 Tg and control animals (*r*^2^ = 0.99 and *r*^2^ = 0.95 for HIV-1 Tg and F344 controls, respectively). The curve was significantly shifted downward [*F*(3,4) = 57.4, *p* ≤ 0.001], however, again, there was no significant shift in the EC_50_; i.e., the clear evidence of a decrease in response vigor as indexed by the decreased overall responding was not accompanied by any evidence for an alteration in efficacy of sucrose as a reinforcer in the HIV-1 Tg animals.

### Sensitivity to, and reinforcing efficacy of, cocaine is diminished in the HIV-1 Tg rats relative to F344 control rats

**Figure 3:**
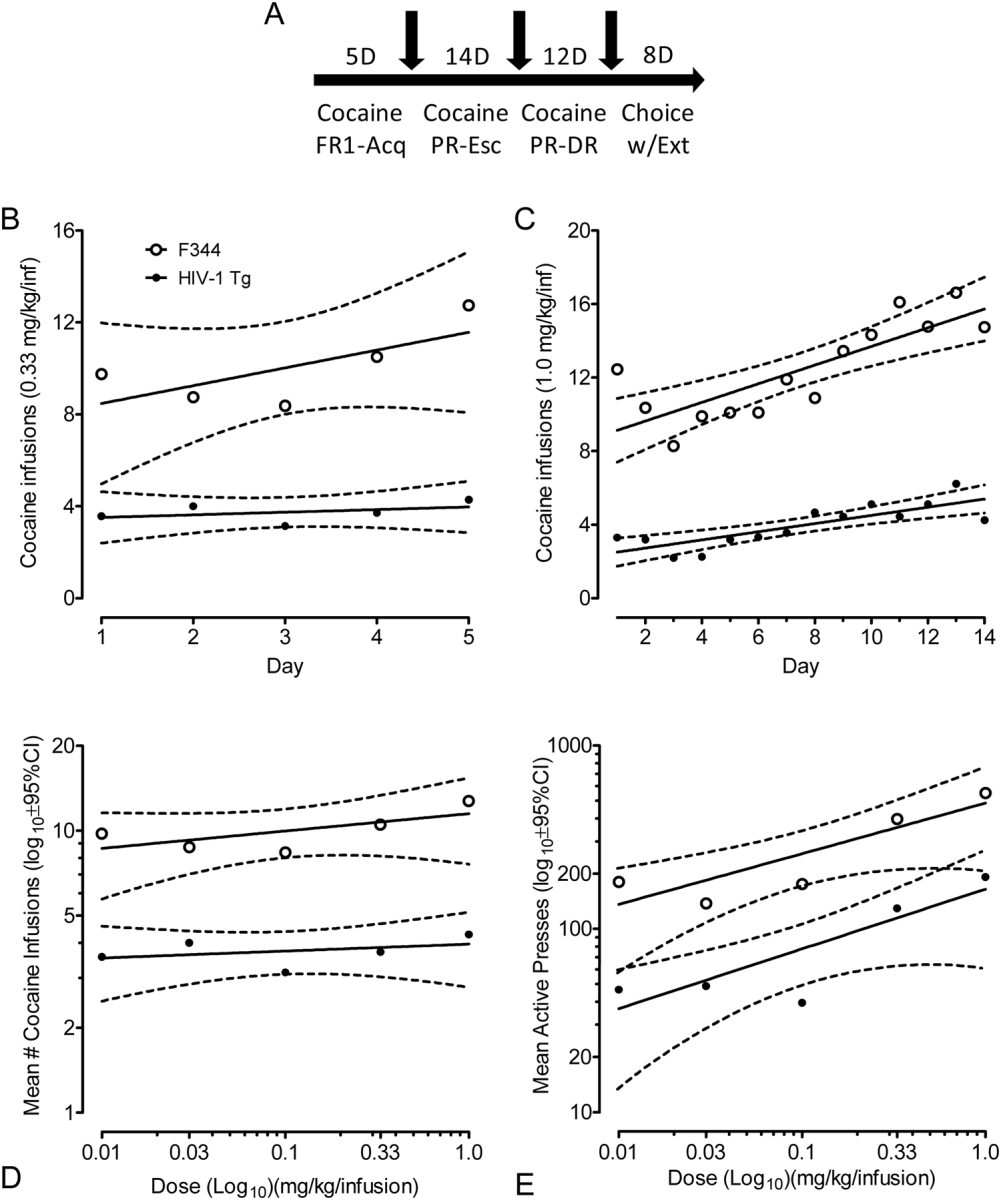
Sensitivity to, and reinforcing efficacy of, cocaine was diminished in the HIV-1 Tg rats relative to F344 control rats. (**A**) Cocaine self-administration (acquisition, escalation, and PR dose-response) was employed to assess potential differences in cocaine-maintained responding between the F344 and HIV-Tg animals. (**B**) The mean number of infusions (±95% CI) earned during the 5-day cocaine acquisition period demonstrated that both HIV-1 Tg and F344 animals maintained a stable response during the acquisition period; however, the HIV-1 Tg animals received significantly fewer infusions than F344 controls [*F*(1,19) = 4.8, *p* ≤ 0.05]. (**C**) The mean number of infusions (±95% CI) earned across the 14-day PR sessions revealed robust effects of genotype [*F*(1,13) = 45.3, *p* ≤ 0.001] and day [*F*(13,169) = 2.9, *p*_GG_ ≤ 0.05], which was functionally linear [*F*(1,13) = 10.0, *p* ≤ 0.01], indicative of an escalation of cocaine intake across sessions. Regression analyses substantiated a prominent linear fit for both genotypes (*r*^2^ = 0.67 and *r*^2^ = 0.66 for F344 and HIV-1 Tg animals, respectively) and further confirmed that the slopes of the lines were significantly different from one another [*F*(1,24) = 6.3 *p* ≤ 0.02]. Escalation to cocaine was significantly slower for the HIV-1 Tg animals relative to F344 controls. (**D**) Number of infusions (±95% CI) earned and (**E**) active lever presses during cocaine dose-response sessions under the PR schedule of reinforcement demonstrated that the HIV-1 Tg animals made significantly fewer active lever presses and received significantly fewer cocaine infusions than the F344 controls. The slopes of the linear log-log fits for number of infusions earned (*r*^2^ = 0.86 and *r*^2^ = 0.45 for F344 and HIV-1 Tg animals, respectively) were significantly different from one another [F(1,6) = 10.1, *p* ≤ 0.02]. Similarly, the slopes of the linear log-log fits for active lever presses (*r*^2^ = 0.90 and *r*^2^ = 0.91 for F344 and HIV-1 Tg animals, respectively) were significantly different from one another [F(1,6) = 9.4, *p* ≤ 0.025]. Thus, sensitivity to, and reinforcing efficacy of, cocaine was diminished in the HIV-1 Tg rats relative to F344 control rats. (n = 10, HIV-1 transgenic F344/NHsd and *n* = 11 F344/NHsd controls for Panels A and B; n = 7, HIV-1 transgenic F344/NHsd and *n* = 8 F344/NHsd controls for Panels C and D).

An FR1 schedule of reinforcement was used to train the animals to self-administer cocaine as well as to assess any potential differences in sensitivity to cocaine as a reinforcer with daily access to a moderate dose of cocaine (0.33 mg/kg/infusion). As illustrated (Fig. 3B), a 2 × 5 repeated-measures ANOVA revealed a significant effect of genotype with HIV-1 Tg animals receiving fewer infusions than F344 controls [*F*(1,19) = 4.8, *p* ≤ 0.05]. There was no significant effect of day nor was there any significant orthogonal component to characterize a trend across days, indicating that the animals neither escalated nor regressed in their drug intake. Furthermore, the response data were significantly greater than zero, suggesting that this dose of cocaine was not aversive to these animals.

Following FR1 training, animals underwent 14 consecutive days of PR responding for a dose of cocaine three times that of the training dose (1.0 mg/kg/infusion). As illustrated (Fig. 3C), a 2 × 14 mixed-design ANOVA on number of cocaine infusions earned indicated a significant effect of genotype [*F*(1,13) = 45.3, *p* ≤ 0.001], and a significant main effect of day [*F*(13, 169) = 2.9, *p*_GG_ ≤ 0.05]. Orthogonal decomposition of the day effect confirmed a prominent linear increase consistent with an escalation of cocaine intake across days [*F*(1,13) = 10.0, *p* ≤ 0.01]. Regression analyses substantiated a prominent linear fit for both genotypes (*r*^2^ = 0.67 and *r*^2^ = 0.66 for F344 and HIV-1 Tg animals, respectively) and further confirmed that the slopes of the lines were significantly different from one another [*F*(1,24) = 6.3 *p* ≤ 0.02]. Thus, differential escalation to cocaine was observed with the HIV-1 Tg animals showing a significantly slower rate relative to F344 controls.

A PR schedule with 5 different concentrations of cocaine was subsequently employed to assess how willing the animals were to work for cocaine with the establishment of their respective dose-response functions. As illustrated (Fig. 3D), a 2 × 5 mixed-design ANOVA on infusions earned found an overall significant effect of genotype with HIV-1 Tg animals earning significantly fewer infusions of cocaine relative to F344 controls [*F*(1,13) = 6.4, *p* ≤ 0.025]. Regression analyses demonstrated a linear log-log fit for both genotypes (*r*^2^ = 0.86 and *r*^2^ = 0.45 for F344 and HIV-1 Tg animals, respectively) and further suggested that the slopes of the lines were significantly different from one another [F(1,6) = 10.1, *p* ≤ 0.02]. Similarly, as illustrated (Fig. 3E), a 2 × 5 mixed-design ANOVA on active lever presses found an overall effect of genotype with HIV-1 Tg animals responding significantly less than controls [*F*(1,13) = 7.4, *p* ≤ 0.02]. Regression analyses confirmed a linear log-log fit for both genotypes (*r*^2^ = 0.90 and *r*^2^ = 0.91 for F344 and HIV-1 Tg animals, respectively) and also suggested that the slopes of the lines were significantly different from one another [F(1,6) = 9.4, *p* ≤ 0.025]. Thus, sensitivity to, and reinforcing efficacy of, cocaine was diminished in the HIV-1 Tg rats relative to F344 control rats.

### Choice between sucrose and cocaine indicated change in preference

**Figure 4:**
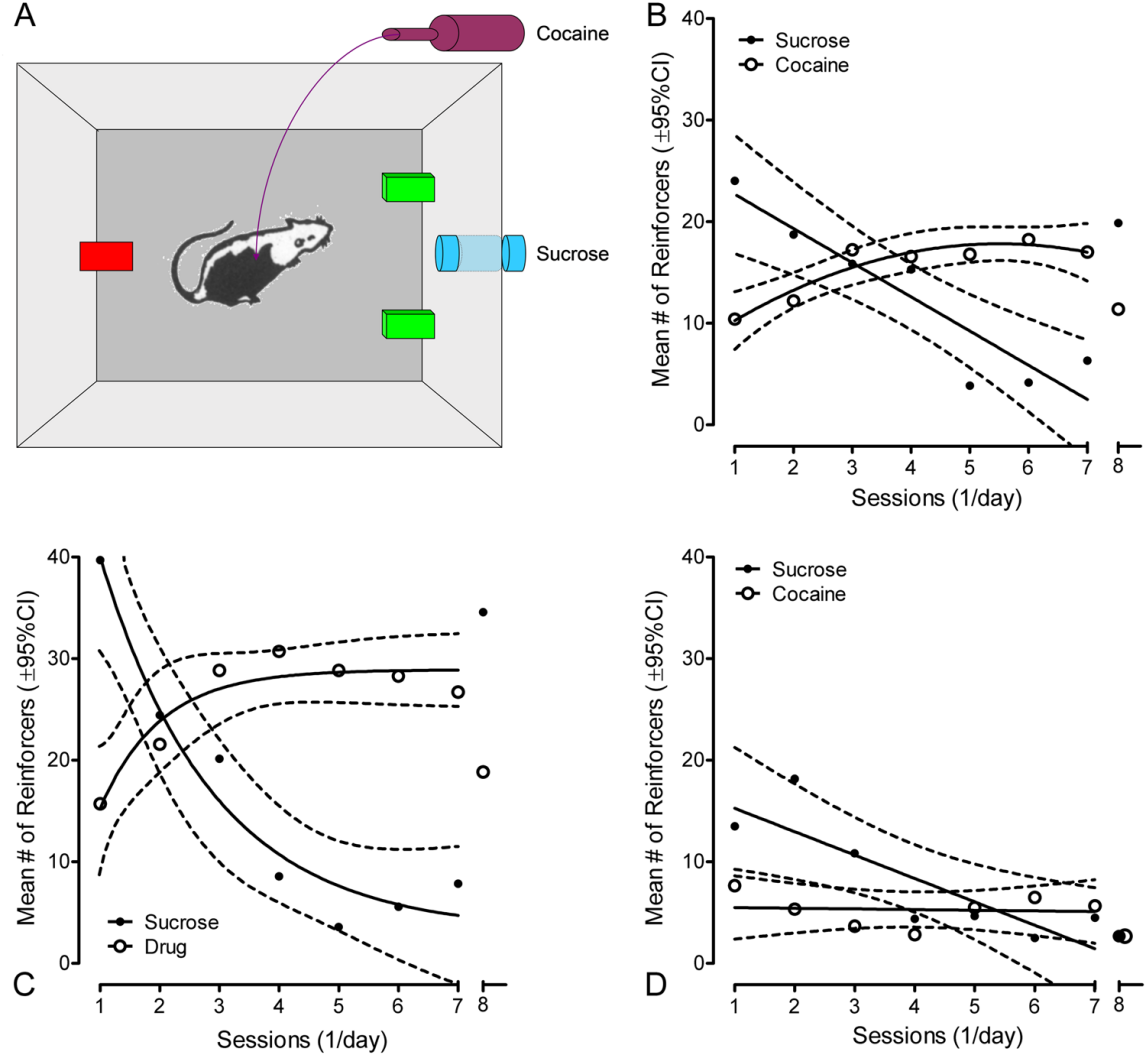
All animals had a history of responding for both sucrose and cocaine, as well as explicit training to preclude the development of a response side bias (diagrammed in **A**). (**B**) The choice behavior of the animals across days, collapsed across genotype, displayed a prominent decrease in sucrose reinforcers earned (linear, *r*^2^ = 0.85) vs. a prominent increase in cocaine infusions earned (single phase association, *r*^2^ = 0.88). The initial choice for sucrose over cocaine by a greater than 2:1 ratio shifted across the 7-day period to a greater than 2:1 ratio for cocaine over sucrose; with the replacement of cocaine with saline on day 8, the drug extinction day, the choice shifted to a return for sucrose over cocaine by an approximate 2:1 ratio. Given the significant genotype × reinforcer type × day interaction [*F*(4,36) = 3.0, *p* ≤ 0.05], the choice behavior of the F344 control group and HIV-1 Tg animals is displayed. (**C**) For the F344 control group, their choice behavior displayed a prominent decrease in sucrose reinforcers earned (single phase decay, *r*^2^ = 0.96) vs. a prominent increase in cocaine infusions earned (single phase association, *r*^2^ = 0.78). The initial choice for sucrose over cocaine by a greater than 2:1 ratio shifted within 5 days to a greater than 2:1 ratio for cocaine over sucrose. With the replacement of cocaine with saline on day 8, the drug extinction day, the F344 control group displayed a significantly greater number of active lever presses for sucrose on day 8 than they did on day 7, the last day of choice, (t(5) = −2.8, *p* ≤ 0.05), and had returned to a level that was not significantly different from the number of active lever presses for sucrose on the first day of the choice period. (**D**) For the HIV-1 Tg group, their choice behavior also displayed a prominent decrease in sucrose reinforcers earned (linear, *r*^2^ = 0.72) vs. only a nominal decrease in cocaine infusions earned (linear, *r*^2^ = 0.008). The initial choice for sucrose over cocaine by a greater than 2:1 ratio shifted across the 7-day period to no choice preference for cocaine vs. sucrose. No rate change was discernible for cocaine infusions earned across the 7-day period (not significantly different from zero, F(1,5) < 1.0]), despite the fact that the HIV-1 Tg animals nevertheless chose cocaine infusions at a mean level greater than zero [F(1,6) = 74.2, *p* ≤ 0.001]. With the replacement of cocaine with saline on day 8, the drug extinction day, no differential choice was observed for the sucrose vs. cocaine response levers. Thus, choice behavior was significantly disrupted by the HIV-1 transgene; i.e., although responding of the HIV-1 Tg animals was sensitive to the reinforcing properties of cocaine, it was not driven by any increase in motivated choice behavior for cocaine. (n = 6, HIV-1 transgenic F344/NHsd and *n* = 7 F344/NHsd controls).

Since the animals had a history of responding for sucrose as well as cocaine, and the animals were also trained to preclude the development of a response side bias, the choice behavior for sucrose vs. cocaine was subsequently assessed (Fig. 4A). Regression analyses on the number of reinforcers earned across days, collapsed across genotype (Fig. 4B), substantiated a prominent decrease in sucrose reinforcers earned (linear, *r*^2^ = 0.85) vs. a prominent increase in cocaine infusions earned (single phase association, *r*^2^ = 0.88). The initial choice for sucrose over cocaine by a greater than 2:1 ratio shifted across the 7-day period to a greater than 2:1 ratio for cocaine over sucrose. The rate of change was −3.4 ± 0.63 for sucrose vs. 1.1 ± 0.35 for cocaine, significantly different from each other [F(1,10) = 38.7, *p* ≤ 0.001] and from zero ([F(1,5) = 28.5, *p* ≤ 0.005 and F(1,5) = 10.3, *p* ≤ 0.025], respectively). With the replacement of cocaine with saline on day 8, the drug extinction day, the choice shifted to a return for sucrose over cocaine by an approximate 2:1 ratio.

The choice behavior of the F344 control group (Fig. 4C) and HIV-1 Tg animals (Fig. 4D) was subsequently examined. The rate of change in choice behavior, captured by a 2 × 2 × 5 repeated measures ANOVA on the number of reinforcers received, revealed a significant genotype × reinforcer type × day interaction [*F*(4,36) = 3.0, *p* ≤ 0.05]. For the F344 control group, regression analyses confirmed a prominent decrease in sucrose reinforcers earned (single phase decay, *r*^2^ = 0.96) vs. a prominent increase in cocaine infusions earned (single phase association, *r*^2^ = 0.78). The initial choice for sucrose over cocaine by a greater than 2:1 ratio shifted within 5 days to a greater than 2:1 ratio for cocaine over sucrose. The rate of change across the 5-day period before reaching their response plateau was −8.8 ± 0.99 for sucrose vs. 3.5 ± 1.08 for cocaine; values significantly different from each other [F(2,6) = 39.8, *p* ≤ 0.001] and from zero ([F(1,3) = 79.5, *p* ≤ 0.005 and F(1,3) = 10.8, *p* ≤ 0.05], respectively). With the replacement of cocaine with saline on day 8, the drug extinction day, the choice shifted to a return for sucrose over cocaine by an approximate 2:1 ratio. The F344 control group displayed a significantly greater number of active lever presses for sucrose on day 8 than they did on day 7, the last day of choice, (t(5) = −2.8, *p* ≤ 0.05), and had returned to a level that was not significantly different from the number of active lever presses for sucrose on the first day of the choice period.

For the HIV-1 Tg group, regression analyses confirmed a prominent decrease in sucrose reinforcers earned (linear, *r*^2^ = 0.72) vs. a nominal decrease in cocaine infusions earned (linear, *r*^2^ = 0.008). The initial choice for sucrose over cocaine by a greater than 2:1 ratio shifted across the 7-day period to no choice preference for cocaine vs. sucrose. The rate of change across the 7-day period was −2.3 ± 0.65 for sucrose vs. −0.07 ± 0.34 for cocaine; values significantly different from each other [F(2,10) = 6.9, *p* ≤ 0.015]. The rate decrease in sucrose reinforcers earned was significantly different from zero ([F(1,5) = 12.7, *p* ≤ 0.025], not unlike that noted in the choice behavior of the F344 control animals. However, no rate change was discernible for cocaine infusions earned across the 7-day period (not significantly different from zero, F(1,5) < 1.0]), despite the fact that the HIV-1 Tg animals nevertheless choose cocaine infusions at a mean level greater than zero [F(1,6) = 74.2, *p* ≤ 0.001]. With the replacement of cocaine with saline on day 8, the drug extinction day, no differential choice was observed for the sucrose vs. cocaine response levers. Collectively, choice behavior was significantly disrupted by the HIV-1 transgene; i.e., although responding of the HIV-1 Tg animals was sensitive to the reinforcing properties of cocaine, it was not driven by any increase in choice behavior for cocaine.

### Dopamine Transporter in the HIV-1 Tg rat

**Figure 5:**
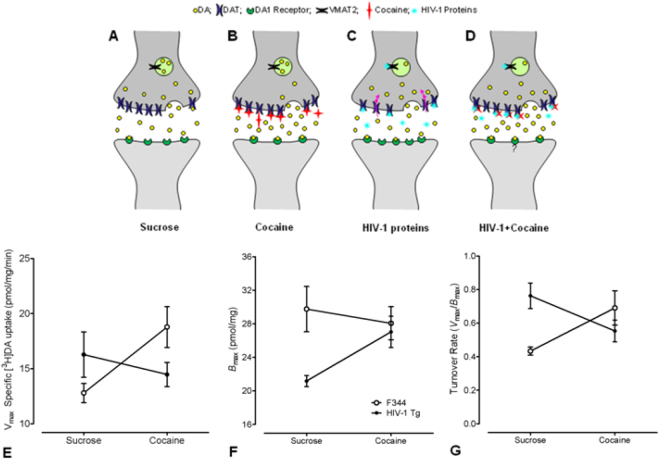
Dopamine transporter dysregulation in HIV-1 Tg animals: sucrose and cocaine self-administration. (n = 12, HIV-1 transgenic F344/NHsd and *n* = 12 F344/NHsd controls; eight each had a history of cocaine, four each only had exposure to sucrose). (**A**,**B**) Illustration of a dopamine terminal/spine following sucrose (**A**) and cocaine (**B**) self-administration in F344 animals. Relative to sucrose self-administration, cocaine self-administration lead to increased rates of dopamine uptake, concentrations and turnover^36–38,69^ and decreased post-synaptic D1 receptors^79^. (**C**) Illustration of a surviving dopamine terminal/spine in HIV-1 Tg animals. HIV-1 proteins (tat, gp120) modulate DAT function^19,20^ and VMAT2^80^. In sum, for the HIV-1 Tg rats, there was an increase in dopamine turnover in the striatum, reflecting that, although there were fewer DAT proteins on the membrane surface, these transporters were more efficient in dopamine clearance in surviving synaptosomes. This may represent a homeostatic process in which surviving synaptic spines of the HIV-1 Tg rat maintain consistent synaptic basal dopaminergic tone. In addition, increased postsynaptic D1 receptors in HIV-1 have been reported^60,81^. (**D**) Illustration of dopamine terminal/spine following cocaine self-administration in HIV-1 Tg animals. Cocaine and HIV-1 proteins both bind to the DAT protein^19^; HIV-1 proteins bind to VMAT2^79^; DAT function is less efficient, resulting in lower turnover rate of dopamine. Dopamine concentrations increase in the presence of both HIV-1 proteins and cocaine, producing a chronic hyperdopaminergic tone^82^. These actions by HIV-1 following repeated cocaine administration may contribute to motivational alterations. Status of D1 postsynaptic receptors is currently unknown. (**E**) [^3^H]DA uptake in striatal synaptosomes from sucrose and cocaine self-administering animals (Mean ± SEM). A significant genotype by treatment interaction was detected *F*(1,20) = 7.8, *p* ≤ 0.01. Cocaine self-administration in F344 animals produced a significant increase in *V*_max_, *p* ≤ 0.05, compared to sucrose F344 controls. There was no significant effect of cocaine self-administration on *V*_max_ in the HIV-1 Tg rat, relative to sucrose; however, cocaine self-administration in HIV-1 Tg animals produced a significantly lower *V*_max_, in comparison to cocaine self-administration in F344 animals, *p* ≤ 0.05. There was no compelling evidence for any effect of genotype, treatment, or a significant interaction, on *K*_m_ in the striatum. All experiments were performed in duplicate, tissue from each animal was considered an individual experiment. (**F**) [^3^H]WIN 35,428 binding to DAT in striatal synaptosomes (Mean ± SEM). HIV-1 Tg sucrose rats had a significantly lower *B*_max_ in comparison to F344 sucrose animals, *F*(1,18) = 4.6, *p* ≤ 0.05. Cocaine self-administration had a significant interaction with genotype *F*(1,18) = 4.2, *p* ≤ 0.05; cocaine self-administering HIV-1 Tg rats increased *B*_max_ to those of cocaine self-administering F344 animals. There was no compelling evidence for any effect of genotype or drug treatment on *K*_d_ in striatal synaptosomes. (**G**) Dopamine turnover rate was determined via *V*_max_/*B*_max_. A significant genotype by treatment interaction was found *F*(1,18) = 6.8, *p* ≤ 0.02. Cocaine self-administration produced a significantly increased turnover rate in F344 animals *p* ≤ 0.05 and, conversely, a significantly decreased turnover rate in HIV-1 Tg animals.

To determine the relationship between brain neurochemistry changes in the DAT and behavior, animals were sacrificed within 12–18 hrs of the last self-administration session. The brains were rapidly dissected and used immediately for neurochemical analyses. These and other relevant neurochemical findings regarding dopamine terminal regulation are summarized in Fig. 5E–G.

### V_max_ increases in F344 controls, but not in HIV-1 Tg animals, following cocaine self-administration

A 2 × 2 ANOVA was used to analyze the effects of cocaine self-administration and genotype on *V*_max_ and *K*_m_ in the striatum. There was a significant genotype by treatment interaction [*F*(1,20) = 7.8, *p* ≤ 0.01] with *V*_max_ as the dependent variable. As illustrated (Fig. 5E), F344 control rats with a history of cocaine self-administration displayed a significantly increased *V*_max_, compared to F344 sucrose rats (*p* ≤ 0.05). Conversely, cocaine self-administration did not significantly affect *V*_max_ in HIV-1 transgenic rats compared to HIV-1 Tg sucrose animals. F344 control animals with a history of cocaine self-administration had significantly higher *V*_max_ rates than HIV-1 Tg animals with a history of cocaine self-administration (*p* ≤ 0.05). There were no significant effects of cocaine self-administration or genotype on *K*_m_ in the striatum.

In the prefrontal cortex, a similar pattern emerged. There was a significant genotype by treatment interaction [*F*(1,20) = 6.1, *p* ≤ 0.02] with *V*_max_ as the dependent variable. HIV-1 Tg animals displayed a significantly reduced *V*_max_ following cocaine self-administration when compared with cocaine naïve HIV-1 Tg rats (*p* ≤ 0.05), while the F344 control animals did not have an altered *V*_max_ following cocaine self-administration. HIV-1 Tg rats with a history of cocaine self-administration displayed a significantly decreased *V*_max_ compared to F344 control rats with a history of cocaine self-administration, *p* ≤ 0.05. There were no significant effects of cocaine self-administration or genotype on *K*_m_ in the PFC.

### B_max_ is significantly decreased in HIV-1 Tg animals and increases following cocaine self-administration

Analysis of maximum binding (B_max_) using a two-way ANOVA revealed a significant main effect of genotype [*F*(1,18) = 4.6, *p* ≤ 0.05]. HIV-1 Tg sucrose rats had a significantly lower maximum [^3^H]WIN 35,428 binding, relative to F344 sucrose animals. Additionally, a significant genotype × treatment (cocaine vs. sucrose) interaction was also detected [*F*(1,18) = 4.2, *p* ≤ 0.05]. As illustrated (Fig. 5F), F344 sucrose control rats had a significantly higher *B*_max_ compared to HIV-1 Tg cocaine naïve rats (*p* ≤ 0.05). *B*_max_ in HIV-1 Tg animals with a history of cocaine self-administration was not significantly different from F344 animals which had also self-administered cocaine. There were no significant differences in *K*_d_ between any of the groups (Mean ± SEM nM: F344-sucrose 18.8 ± 2.2, F344-cocaine 14.5 ± 0.9, HIV-1 Tg-sucrose 16.1 ± 1.5, HIV-1 Tg-cocaine 15.0 ± 0.9).

### Decreased DA turnover rate in the striatum of the HIV-1 Tg rat following cocaine self-administration

DA turnover rate was determined by dividing striatal *V*_max_ by striatal *B*_max_. Using the resulting turnover values, a two-way ANOVA determined effects of cocaine self-administration on DA turnover in these animals. Although no main effects of genotype or treatment were found, a significant genotype × treatment interaction was noted [*F*(1,18) = 6.7, *p* ≤ 0.02]. As illustrated (Fig. 5G), F344 animals had a significantly increased DA turnover rate following cocaine self-administration (*p* ≤ 0.05); in contrast, HIV-1 Tg animals showed a significant decrease in DA turnover following cocaine self-administration (*p* ≤ 0.05).

### Behavior predicting neurochemical outcomes: Cocaine and sucrose

#### Cocaine

The cocaine FR acquisition period was determined to significantly predict *V*_max_ in the PFC (data from each day were considered to be independent predictors, [*F*(5,8) = 12.3, *p* ≤ 0.005]). The adjusted *r*^2^ indicated that 81.4% of the variance in PFC *V*_max_ was associated with number of infusions received on days 1–5 of cocaine acquisition. Cocaine self-administration produced a significant decrease in [^3^H]DA *V*_max_ in the HIV-1 Tg rat PFC in comparison to both HIV-1 Tg drug-naïve animals and F344 cocaine self-administration animals. In contrast, cocaine self-administration did not significantly affect [^3^H]DA *V*_max_ in the PFC of F344 animals, which is similar to previous findings^36–39^. Interestingly, the number of cocaine infusions received during cocaine acquisition significantly predicted the neurochemical changes in the PFC, suggesting that DA in the PFC plays a significant role in establishing cocaine reinforcement in the HIV-1 Tg rat.

The cocaine PR escalation period was also determined to significantly predict *V*_max_ in the PFC using multiple linear regression (data from each day – day 1 to day 14–were considered to be independent predictors, [*F*(7,4) = 20.6, *p* ≤ 0.01]). The adjusted *r*^2^ indicated that 92.6% of the variance in PFC *V*_max_ was associated with the number of lever presses across the 14-day period of cocaine escalation. The number of active lever presses during the cocaine PR dose-response experiment (data from each dose were considered independent predictors) was determined to significantly predict both *V*_max_ in the striatum [*F*(5,6) = 15.8, *p* ≤ 0.005] and DA turnover [*F*(5,6) = 17.1, *p* ≤ 0.005]. The adjusted *r*^2^ values indicated that 87% of the variance in STR *V*_max_ and 88% of the variance in DA turnover were associated with the number of active lever presses during the cocaine PR dose-response experiment. The number of infusions during the cocaine PR dose-response experiment (data from each dose were considered independent predictors) was determined to significantly predict *B*_max_ in the striatum [*F*(5,6) = 6.4, *p* ≤ 0.05]. The adjusted *r*^2^ indicated that 71.3% of the variance in STR *B*_max_ was associated with the number of infusions received during the cocaine PR dose-response experiment. Thus, those neurochemical measures that were significantly predicted by behavioral measures were PFC *V*_max_, STR *V*_max_, STR *B*_max_, and DA turnover in multiple regression analyses.

#### Sucrose

FR responding for sucrose did not predict neurochemical outcomes. The number of active lever presses during the sucrose PR dose-response experiment (data from each concentration were considered independent predictors) was determined to significantly predict both *V*_max_ in the striatum [*F*(6,15) = 3.0, *p* ≤ 0.05] and DA turnover in the striatum [*F*(6,15) = 3.1, *p* ≤ 0.05] using multiple linear regression. The adjusted *r*^2^ values indicated that 35.7% of the variance in STR *V*_max_ and 37.9% of the variance in DA turnover were associated with the number of active lever presses during the sucrose PR experiment. The slope of the linear regression lines representing the change in sucrose preference over the first 5 days of the choice period significantly predicted *B*_max_ in the striatum in the control group [*F*(1,4) = 8.3, *p* ≤ 0.05], but not in the HIV-1 Tg group. The adjusted *r*^2^ indicated that 59.6% of the variance in STR *B*_max_ was associated with the change in sucrose preference over the 5-day period. Relative to the number of significant behavioral:neurochemical correlations found for cocaine reinforcement, far fewer behavioral alterations for sucrose reinforcement predicted alterations in dopamine neurochemistry.

## Discussion

Motivational alterations in HIV-1+ individuals are associated with decreased performance on tasks known to involve frontal-subcortical circuitry^21^, and with decreased volume of the NAc^22^. The alterations in reinforcing efficacy found in the HIV-1 Tg rat resembles altered motivational states following HIV-1 infection^1,3^. These alterations may reflect selective dysregulation within dopaminergic circuitry by HIV-1 proteins. DAT function was altered in the HIV-1 Tg rats; prior cocaine self-administration produced a unique effect on dopamine homeostasis. Specifically, although both HIV-1 Tg and control animals increased cocaine intake over 14-days of PR responding, the HIV-1 Tg rats responded significantly less and maintained consistent low-level responding to cocaine (unit dose 1.0 mg/kg/infusion). There was no significant shift in EC_50_ for sucrose, although HIV-1 Tg rats responded significantly less for sucrose using both FR-1 and PR schedules of reinforcement. These findings suggest a profound effect on cocaine-maintained responding in the HIV-1 Tg rats. When control animals were allowed a choice between cocaine and sucrose, they initially preferred sucrose, but over time switched to a preference for cocaine. HIV-1 Tg rats exhibit disrupted choice behavior with no preference for cocaine, further suggesting a dysregulation of motivation.

HIV-1 proviruses in the brain produce viral proteins, and the production of these HIV-1 proteins is, for the most part, unaffected by antiretroviral drugs. HIV-1 patients with latent HIV-1 infection (HIV-1+ without HIV-1 viral RNA or p24) may display mild to moderate cognitive impairment^16^. HIV-1 Tat protein is found in the CSF of aviremic patients^17^. Constitutive HIV-1 protein expression in the HIV-1 Tg rat is under the control of the natural HIV-1 promoter, LTR, with HIV-1 protein expression in mononuclear phagocytes^25^, astrocytes and possibly other cells^26^. The expression of HIV-1 protein mRNA (Tat, gp120, nef and vif mRNA) is not uniformly distributed across brain regions, but is concentrated in the striatum and cortex, with lowest mRNA expression in the hypothalamus and hippocampus^40^. The gp120 protein has been found in HIV-1+ humans early in the pandemic^41,42^ and gp120 exposure results in dopamine neuropathology *in vitro*^20,43^ and *in vivo*^44^. Thus, the HIV-1 Tg rat provides a chronic exposure of specific brain regions to low levels of HIV-1 viral proteins under the control of the natural promoter, and although the expression levels of the proteins may vary in different regions and cell types, these proteins are generated from integrated proviral DNA (without active infection). HIV-1 protein expression in the transgenic rat predominates in the basal ganglia and cortical regions, similar to the brain regional HIV-1 viral load distributions in humans^45^.

Given that the HIV-1 Tg rat is non-infectious, it is not used to study the effects of HIV-1 viral replication, HIV-1 infection, or  the effects of cART on viral replication^46^. However, the HIV-1 T rat may be used to study the long-term effects (months to years) of low-level viral protein exposure^40,47^. In particular the HIV-1 Tg rat may help understand neurologic dysfunction in HIV-1 infected individuals in the post-cART era, in which peripheral viral replication is well-controlled. HIV-1 Tg young adult rats are generally healthy^48^, growing at similar rates as F344 controls^49^, and have been shown to perform operant executive function tasks^50^. The general health of the HIV-1 Tg rat permits longitudinal study of behavioral/functional impairments over a prolonged period, which is key in understanding progressive disorders of neurocognition, as expressed in HAND and motivational alterations. Two prior studies^51,52^ demonstrated that the HIV-1 Tg rat was capable of intravenous self-administration of cocaine, and that cocaine served as a reinforcer in these animals.

We found that cocaine, but not sucrose, had a lower reinforcing efficacy in the HIV-1 Tg rat. First, we used the sucrose preference task to assess changes in gustatory perception, and similar to a prior report regarding taste^40^, we found no difference in sucrose preference between HIV-1 Tg rats and controls. Second, HIV-1 Tg rats consistently responded for fewer sucrose reinforcers when compared to control rats, although critically there was no shift in the EC_50_ between groups. It is important to note that the animals were neither food nor water restricted during PR testing, so responses for sucrose were not affected by variations in satiety. Relative to mechanisms of cocaine reinforcement, such as DAT function, alterations in sucrose reinforcement may reflect an impairment in either more widespread neural pathways^53^ or dysfunction of a separate, discrete, neuronal system located within the NAc which is sensitive to sucrose and insensitive to cocaine^54,55^. As we found no shift in the EC_50_ for sucrose relative to controls, the neuronal systems underlying sucrose reinforcement may be less impaired in the HIV-1 Tg rats, relative to those for cocaine reinforcement.

It is interesting to compare the findings from the PR test with the choice task, as the two procedures may not assess the same motivational processes^56^. All animals had extensive experience with both cocaine and sucrose reinforcement and were explicitly trained such that each lever had an equal likelihood of presenting either reinforcer (i.e., no bias). Importantly, cocaine preference was not attributed to a lack of interest in sucrose. Prior to the choice procedure, both cocaine and sucrose were consumed on several trials in which each reinforcer was presented alone. We found in the choice procedure, animals initially had a strong preference for choosing sucrose over cocaine. This is consistent with prior publications in which cocaine was found to have low value relative to natural rewards, such as sucrose, in rats^57^. Interestingly, over a period of days, the control animals shifted to a cocaine preference, suggesting an addictive process in that animals ultimately preferred to take cocaine at the expense of other available choices (i.e., sucrose); however, choice behavior was disrupted for the HIV-1 Tg rats. The HIV-1 Tg animals were sensitive to the reinforcing properties of cocaine (as indicated by saline substitution), but there was no increase in choice behavior for cocaine, suggesting a motivational deficit independent of alterations in reward. Dopamine, and in particular the DAT, within the NAc is a vital component of the circuitry which is thought to underlie choice and apathetic behavior^58,59^.

The direct modulation of HIV-1 Tat protein binding onto the DAT has been proposed as a therapeutic target for HAND; however, there are significant inconsistencies reported in DAT functional alterations following HIV-1 exposure. First, using *in vitro* models of HIV-1 Tat - DAT protein:protein interactions we have consistently found Tat-induced *reductions* in DAT function using either midbrain neurons in culture^60^, purified Tat/applied to control synaptosomal preparations^19,20^ or direct infusion of Tat protein into brain tissue^61^. Second, in direct contrast, a prior report using synaptosomes prepared from HIV-1 Tg rat brain tissue reported *increased* DAT activity^62^. In order to determine the basis for this discrepancy, we recently examined whether DAT activity was increased or decreased in the HIV-1 Tg animals using an *ex vivo* striatal slice preparation^63^. We reported that striatal brain slices from HIV-1 Tg rats had clear decreases in DAT function. Additionally, recent *in vivo* PET imaging of dopaminergic systems in HIV-1 Tg rats also found decreased presynaptic DAT^64^, which is consistent with human PET studies of DAT in HIV-1 patients^65^. Given the directly contrasting results reported between decreased DAT activity in striatal slices/imaging of HIV-1 Tg rats vs. the increased DAT activity in striatal synaptosomes prepared from HIV-1 Tg rats, it appears that the increased DAT activity is isolated to the use of synaptosomal preparations. In contrast to synaptosomal preparations, brain slice preparations maintain the *in vivo* synaptic architecture^66^, reflecting pre- and post-synaptic and local circuit integrity.

We have reported increased populations of abnormal short, stubby, spines in HIV-1 Tg rats^48^; synaptosomes are prepared from only non-stubby spines of HIV-1 Tg rats. These fully functional (i.e., non-stubby) synaptosomes may reflect an increased, compensatory, response to prolonged exposures to HIV-1 proteins and/or excess dopamine concentrations. Thus, HIV-1 Tg rat synaptosomes may be reflective of long-term exposure to HIV-1 proteins, reflecting an increase in DAT activity, which is mechanistically distinct from *in vitro* exposure to purified Tat protein^19^ in which DAT function is attenuated. Therapeutic strategies directed to the DAT in neuroHIV-1+ may need to 1) incorporate a longitudinal *in vivo* model incorporating both structural and functional measures, and 2) target therapeutic approaches to increasing circuit synaptic/spine plasticity – rather than attempting to further increase DAT function which is already maximized in surviving spines. Extrapolation of acute studies of DAT:Tat protein interactions may lack translational relevance in HAND. Nevertheless, unique insights may be gained by examining synaptosomes, especially regarding the pathophysiology and compensatory responses of synapses/spines in chronic neurological diseases^67^ and neurocognitive impairments^68^.

Dopamine homeostasis was differentially affected by cocaine self-administration in the HIV-1 Tg rats. Similar to previous findings, cocaine self-administration increased V_max_ in control rats^69^; however, cocaine self-administration did not significantly increase V_max_ in HIV-1 Tg animals. Although cocaine self-administration resulted in an increase in *B*_max_ in HIV-1 Tg rats, the lack of change in V_max_ resulted in a low DA turnover rate in cocaine -HIV-1 Tg rats, further highlighting dopaminergic dysfunction in the striatum. One important caveat to the current studies is the differential intake of both sucrose and cocaine between HIV-1 Tg and control groups in the study of motivational differences, which may influence post-mortem brain measures. However, a prior study of cocaine self-administration in HIV-1 Tg rats^51^ similarly reported an increase in striatal DAT binding sites, without any differences in lifetime cocaine intake between HIV-1 Tg and control groups. Collectively, HIV-1 may contribute to decreased DA turnover, but only when challenged via prior cocaine experience, possibly as a compensatory response to cocaine-induced increases in extracellular concentrations of dopamine.

The nucleus accumbens/ventral striatum decreases in volume following chronic HIV-1 infection^22^ and is a key area for reinforcing actions^70^. In particular, the medium spiny neurons (MSNs) of the NAc play a key role in many motivational^71^ and goal-directed behaviors^72^. We have reported that the MSNs of the NAc are morphologically altered in HIV-1 Tg female rats^48^, with an increase in short, stubby, spines - which are generally regarded as inactive or less plastic with stubby spines having reduced synaptic contact area, relative to longer spines^73^. The MSN spine neck receives approximately 70% of dopaminergic synapses^74^. Thus, an increase in stubby spines reduces the MSN contact area for dopaminergic afferents from the ventral tegmental area. MSNs also receive regulatory input from the prefrontal cortex^75^, which is also altered in HIV-1 Tg animals^76^. It is possible that the MSNs represent a structural loci (or part of key circuitry) for the actions of HIV-1 proteins on goal directed behaviors. Effective therapeutic approaches for apathy might be directed to therapeutics (i.e. phytoestrogens, S-equol;^28,77^) for increasing spine and circuitry connectivity (VTA-NAc-PFC) more generally in HIV-1 Tg rats, rather than focusing on a restrictive targeting of the DAT.

In sum, HIV-1 proteins produce unique long-term motivational and neurochemical outcomes which may contribute to apathy prominent in HIV-1+ patients. A dysregulation of motivation was suggested by the disruptions of choice behavior, with no preference for cocaine as well as reduced response vigor for sucrose by the HIV-1 Tg rats. Accompanying these behavioral changes, we found that HIV-1 produces neurochemical alterations in DAT function. Over a prolonged period of exposure to HIV-1, loss of dopaminergic nerve terminals^11^, shorter and less plastic dendritic spines^48,76,77^ and loss of dopamine may occur, as has been reported in HIV-1+ human drug abusers^78^. Our results suggest that DA circuitry dysfunction precipitated by HIV-1+ underlies the alterations of goal-directed behaviors. Enhancing synaptic plasticity and circuit functionality in the presence of HIV-1 may lead to effective and uniquely targeted therapeutics for motivational dysregulation and apathy in HIV-1+ individuals.

### Data Availability Statement

All relevant data are within the paper.
